# Climatic differentiation in polyploid apomictic *Ranunculus auricomus* complex in Europe

**DOI:** 10.1186/s12898-018-0172-1

**Published:** 2018-05-21

**Authors:** Juraj Paule, Franz G. Dunkel, Marco Schmidt, Thomas Gregor

**Affiliations:** 1 Department of Botany and Molecular Evolution, Senckenberg Research Institute and Natural History Museum, Senckenberganlage 25, 60325 Frankfurt am Main, Germany; 2 Department of Diversity, Evolution and Phylogeny of Higher Plants and Lichens, Institute for Ecology, Evolution and Diversity, Goethe University, Max-von-Laue-Str. 13, 60438 Frankfurt am Main, Germany; 3 Am Saupurzel 1, 97753 Karlstadt, Germany; 4 Data and Modelling Centre, Senckenberg Biodiversity and Climate Research Centre (BiK-F), Senckenberganlage 25, 60325 Frankfurt am Main, Germany; 5 Scientific Service, Palmengarten, Siesmayerstraße 61, 60323 Frankfurt am Main, Germany

**Keywords:** Apomixis, Chromosome number, Distribution, Flow cytometry, Ploidy, Postglacial colonization, Relic area, Species distribution modelling, Ranunculaceae

## Abstract

**Background:**

Polyploidy and apomixis are important factors influencing plant distributions often resulting in range shifts, expansions and geographical parthenogenesis. We used the *Ranunculus auricomus* complex as a model to asses if the past and present distribution and climatic preferences were determined by these phenomena.

**Results:**

Ecological differentiation among diploids and polyploids was tested by comparing the sets of climatic variables and distribution modelling using 191 novel ploidy estimations and 561 literature data. Significant differences in relative genome size on the diploid level were recorded between the “*auricomus*” and “*cassubicus*” groups and several new diploid occurrences were found in Slovenia and Hungary. The current distribution of diploids overlapped with the modelled paleodistribution (22 kyr BP), except Austria and the Carpathians, which are proposed to be colonized later on from refugia in the Balkans. Current and historical presence of diploids from the *R. auricomus* complex is suggested also for the foothills of the Caucasus. Based on comparisons of the climatic preferences polyploids from the *R. auricomus* complex occupy slightly drier and colder habitats than the diploids.

**Conclusions:**

The change of reproductive mode and selection due to competition with the diploid ancestors may have facilitated the establishment of polyploids within the *R. auricomus* complex in environments slightly cooler and drier, than those tolerated by diploid ancestors. Much broader distribution of polyploid apomicts may have been achieved due to faster colonization mediated by uniparental reproductive system.

**Electronic supplementary material:**

The online version of this article (10.1186/s12898-018-0172-1) contains supplementary material, which is available to authorized users.

## Background

Multiplication of the whole chromosome set, also referred to as polyploidy is considered one of the most important drivers of plant evolution, in particular when considering sympatric and parapatric speciation [1]. Polyploidization has instant and tremendous consequences for the whole genome structure, creating variation by, e.g., novel allelic combinations, gene dosage, heterosis, regulatory interactions or altered epigenetics [2–4]. After successful establishment polyploids are thought to be advantageously (pre-)adapted to more extreme climates than diploids as they are usually found in comparatively harsher environments (e.g., arid, arctic, alpine or artificially disturbed habitats) [5, 6].

Polyploidy is also considered to play an important role in plant diversification during Pleistocene glacial cycles. Stebbins’ secondary contact hypothesis [5] explains the origin of polyploids by climate induced range fragmentation and repeated contacts of diploid lineages in glacial refugia, resulting in hybridization and subsequent stabilization by chromosome doubling (allopolyploidy). As a consequence of novel allelic combinations resulting in altered gene expression, polyploids may exhibit higher fitness [7], enhanced niche breadth [8] and range expansions [9]. Tightly linked to polyploidy is also apomixis (asexual reproduction through seeds), which might fix the novel and possibly advantageous allelic combinations over several generations [10]. Empirical observations indicate that apomicts inhabit marginal and disturbed habitats (but see [11]), which is why apomixis is also considered to play an enhancing role in colonization and range expansions [10]. Superior colonizing potential of apomicts, often referred to as “geographical parthenogenesis”, is partially explained by population establishment through single seed/individual (Baker’s law), [11–14].

The *Ranunculus auricomus* complex is a suitable model to study simultaneous effects of the above-mentioned phenomena. The complex comprises ca. 900 polyploid apomictic and four diploid sexual taxa and occurs in mesophilic deciduous forests throughout Europe, Siberia and the Caucasus as well as in Iceland and Greenland [15–17]. Polyploidy is almost always correlated with facultative pseudogamous apospory (apomixis), which requires fertilization for endosperm development [18–22]. However, rarely also autotetraploid sexual lineages have been recorded [23–25]. It has been suggested that the morphological diversity of the apomictic complex originates from hybridizations of morphologically divergent sexual ancestors followed by polyploidization and that relationships within the complex are highly reticulate [15, 26–28]. The morphological variation is however discontinuous and four groups are traditionally distinguished based on the leaf morphology [15, 26, 27, 29]: “*auricomus*”, “*cassubicus*”, “*fallax*” and “*monophyllus*”. Sexual taxa have been recorded only in the “*auricomus*” and “*cassubicus*” groups, whereas allopolyploids from all four groups are regarded as morphological intermediates between the sexual taxa. It has been estimated that sexuals from the “*auricomus*” and “*cassubicus*” groups separated ca. 900 kyr BP (thousand years before present), whereas apomictic allopolyploids are thought to have arisen during the last glacial period (115–15 kyr BP) as a result of secondary contacts of diploid progenitors due to range fragmentation and expansions as a consequence of climatic changes [25, 30, 31]. Interestingly, habitat differentiation on a microscale and a tendency to inhabit artificial meadows is more pronounced in apomictic than in sexual populations [32]. The complex as a whole shows geographical parthenogenesis, with polyploid apomicts covering the whole distribution range and with sexual taxa restricted to few geographically isolated areas [15, 18, 33].

Even though there has been ample research on the *R. auricomus* complex in the last decades, certain parts of the distribution and taxa were not yet studied in detail. The hypothesis of geographical parthenogenesis and ecological differentiation among diploids and polyploids was not yet addressed on a continental scale. In order to specifically test these patterns we combine newly acquired flow cytometric ploidy estimations from Europe with published georeferenced chromosome numbers and ploidy estimations and aim to answer the following questions:

i. Are there differences in relative genome size between diploid lineages in the morphologically defined groups “*auricomus*” and “*cassubicus*”?
ii. What is the geographical and ecological distribution of different ploidy levels of the *Ranunculus auricomus* complex in Europe? How strong is the climatic differentiation between diploids and polyploids?
iii. Does the geographical distribution of diploid taxa reflect the presence of the glacial refugia?

## Methods

### Plant material

In total, 191 individuals covering 136 taxa were collected covering most of the European distribution range and major taxonomic groups of the *R. auricomus* complex (“*auricomus*”, “*cassubicus*” and “*fallax*” groups). The sampling was designed based on herbarium survey with the special focus on the taxa and localities with unknown ploidy. Herbarium vouchers of studied accessions are deposited in the herbarium of the Botanische Staatssammlung München (M), as well as in the private herbarium of the second author. Detailed collection history is given in Additional file 1. The geographic coordinates of studied accessions were recorded in WGS84 coordinate system using a hand-held GPS device. In order to present the geographical data ArcGIS/ArcMap v10.1 (ESRI, Redlands, California, USA) software was used. Although the division into groups is difficult in polyploids due to reticulate evolutionary background we keep it for sake of clearer presentation of data. Material collected within this study was assigned to groups based on size, the presence of cataphylls, number and division of the basal leaves [34]. Due to few published records (21 counts from Siberia and Central Asia) the predominantly Asian “*monophyllus*” group was not considered in our study. *R. allemannii*, a widespread species of the Central Alps, is often regarded as a member of the “*monophyllus*” group [29, 34, 35]. However, due to morphological affinities (i.e. mostly divided first basal leaf and broadly oblanceolate stem leaves) we treat *R. allemannii* as a member of the “*cassubicus*” group [36, 37], a relationship that is also supported by ITS data [38].

### DNA ploidy estimation

DNA-ploidy levels were estimated by flow cytometry of fresh leaves using a Partec CyFlow space (Partec, Münster, Germany) fitted with a high power UV LED (365 nm). Leaf tissues of the analysed sample and internal standard *Pisum sativum* cv. Ctirad (2C = 9.09 pg [39]) were co-chopped using a razor blade in a plastic Petri-dish containing 1 ml of ice-cold Otto I buffer (0.1 M citric acid, 0.5% Tween 20; [40]). The suspension was filtered through Partec CellTrics^®^ 30 µm to remove tissue debris and incubated for at least 10 min at room temperature. Isolated nuclei in filtered suspension were stained with 1 ml of Otto II buffer (0.4 M Na_2_HPO_4_ × 12H_2_O) containing the AT-specific fluorochrome 4′,6-diamidino-2-phenylindole (DAPI; 4 µg ml^−1^) and β-mercaptoethanol (2 µg ml^−1^). The relative fluorescence intensity was recorded for 3000 particles. Sample/standard fluorescence ratios (relative genome size) were calculated from the means of fluorescence histograms visualized using the FloMax v2.4d software (Partec). Only histograms with coefficients of variation (CVs) ≤ 5% for the G_0_/G_1_ peak of the sample were considered. Seven chromosome counted individuals of different ploidy (Additional files 1 and 2) served as reference for the DNA ploidy estimation. The DNA-ploidy was attributed based on the regression of relative genome sizes of the counted individuals.

### Calibration chromosome counts

Root tips of seven individuals were pre-treated with 0.1 mM 8-Hydroxyquinoline for 4 h at room temperature and fixed in ice-cold 3:1 ethanol:acetic acid for 4 h. Until further analysis the root tips were stored in 100% ethanol. Maceration lasted for 10 min in concentrated HCl at room temperature. The tissue was subsequently squashed in a drop of aceto-orcein. Chromosomes were counted using a light microscope Leica DM 1000 LED (Leica, Wetzlar, Germany) with a 10 × 100 magnification.

### Literature review

For the review of previously published chromosome numbers we collected 561 chromosome counted specimens from literature (Additional file 3) and georeferenced them with a spatial precision of at least 10 km. If possible, counts were attributed to one of the three groups of the *R. auricomus* complex mentioned above.

### Climatic characterization of habitats

In order to explore potential influence of the climatic conditions on the distribution patterns, climatic variables were extracted from WorldClim v1.4 [41] and tested for their ability to differentiate between ploidy levels within studied groups. Distribution data (occurrence points) from the studied material were assembled with the data retrieved from the literature review and 19 climatic variables (Bio1‒Bio19) were extracted from the WorldClim dataset with a resolution of 30 arc-seconds (i.e. approximately one square kilometer). In order to reduce spatial bias in the distribution modelling (see below), the records have been subsampled to include only a single record (the one closest to the center) within a 0.1° grid cell (c. 10 km × 10 km).

### Data analyses

Statistical computations were performed in R v3.2.2 [42]. Due to reticulate evolutionary background of polyploids, analyses were carried out with following groups: diploids from the “*auricomus*” and “*cassubicus*” groups, all diploids and all polyploids. Differences in relative genome sizes between diploids from the “*auricomus*” and “*cassubicus*” groups as well as between climatic preferences of ploidy levels (diploid vs polyploid) were assessed by a non-parametric Wilcoxon rank-sum test (= Mann–Whitney U test) due to the violation of normal distribution of the data (Shapiro–Wilk test). The data were visualized using boxplots and stripcharts. Climatic preferences of diploids and polyploids were also compared using principal component analysis (PCA) using “dudi.pca” from the R package ade4 v1.4-14 [43] based on a correlation matrix. For variable pairs with absolute correlation coefficients higher than 0.8 (Additional file 4) only one, biologically more significant variable, was kept (Additional file 5). Statistical differences between diploids and polyploids were additionally assessed using the non-parametric Wilcoxon rank-sum test by comparing the principal components (PC1, PC2, PC3).

### Distribution modelling

Distribution models were applied for diploids from the “*auricomus*” and “*cassubicus*” groups and all polyploids, using MaxEnt v3.3.3 [44] with climatic variables from WorldClim v1.4 as predictors. The climate layers were cut to a rectangle around the occurrences ranging from 35°N to 73°N and 37°W to 46°E. Ten variables were used for modelling after removal of the biologically less significant variables from variable pairs with absolute correlation coefficients higher than 0.8 (Additional files 4, 5). For projections into the past, we used WorldClim’s paleoclimate layers (Last Glacial Maximum (LGM), 22 kyr BP) for three different Global Climate Models (CCSM4, MIROC-ESM, MPI-ESM-P) for the LGM at a resolution of 2.5’. Occurrence points have been assigned to the ploidy level (di- vs polyploid) as well as to the taxonomic groups, in which both ploidy categories occur (“*auricomus*”, “*cassubicus*” groups). We removed duplicate records, reserved 25% of occurrence points for testing, enabled all features in MaxEnt and used the median out of 10 model runs. For evaluation of the distribution models, we used the AUC (area under the model’s receiver-operator-characteristic curve) [45].

## Results

### DNA ploidy estimation and literature review

**Fig. 1:**
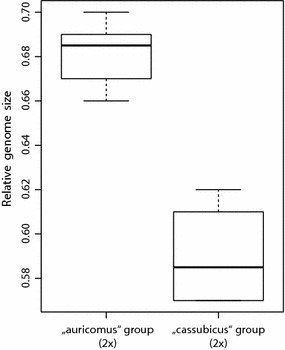
Boxplots of relative genome sizes of diploids from the “*auricomus*” and “*cassubicus*” groups. Relative genome size is expressed as a ratio of the sample and the internal reference standard (*P. sativum*)

The DNA-ploidy was determined for 191 individuals from 163 localities covering ca 95 taxa with previously unknown ploidy (Additional file 1). The CVs of the G_0_/G_1_ sample peaks ranged from 0.96 to 5.04 (mean 2.34 ± 0.85). Five distinct classes of relative genome sizes were detected. These corresponded to di- tri-, tetra-, penta- and hexaploidy (Table 1, Additional files 1 and 2) and were confirmed by seven chromosome-counted individuals (2n = 2× = 16: Du-27660-1, Du-30442-1; 2n = 3× = ca 25: Du-28580; 2n = 4× = 32: Du-12533, Du-27585, 2n = 4× = ca. 32: Du-21045, Du-27367; Additional file 2). The distribution of the relative genome sizes comprising the whole sampling is shown in Additional file 2. On the diploid level significant differences in relative genome size were observed between “*auricomus*” (mean ± SD: 0.68 ± 0.01) and “*cassubicus*” (0.59 ± 0.02) group (Wilcoxon rank sum test, W = 84, p < 0.01; Fig. 1).

**Table 1:** Relative genome size and derived DNA ploidy of studied groups from the *R. auricomus* complex

Group	No samples	Relative genome size ± SD	DNA ploidy
“*auricomus*” group	14	0.68 ± 0.012	2×
	1	1.00	3×
	122	1.34 ± 0.049	4×
	2	1.93 ± 0.011	6×
“*cassubicus*” group	6	0.59 ± 0.022	2×
	15	1.24 ± 0.099	4×
	2	1.60 ± 0.005	5×
“*fallax*” group	1	1.03	3×
	27	1.30 ± 0.062	4×
	1	1.90	6×

Literature review revealed 561 counts covering all three studied groups. For the “*auricomus*” group 308 chromosome counts were recovered, for the “*cassubicus*” group 137, for the “*fallax*” group 68, and 48 chromosome counts could not be attributed due to ambiguous taxonomic and locality assignments (Additional file 3). Chromosome counts of 2n = 44 by Jankun and Izmaiłow [46] are interpreted as hexaploid.

Considering both published and newly acquired data (Additional files 1 and 3) tetraploids were the most common cytotypes (80.6%), followed by diploids (6.9%), hexaploids (5.9%) and other minority cytotypes. The combined ploidy dataset cover approximately 300 taxa of the complex and provide good representation of European distribution except localities from Ukraine und European Russia. The “*auricomus*” group comprised di-, tri, tetra-, penta-, hexa-, and heptaploids, the “*cassubicus*” group di-, tri-, tetra-, penta-, hexa- and octaploids and within the “*fallax*” group tri-, tetra-, hexa- and a pentaploid were recorded. One ploidy level was found in most of the species except for *R. marsicus* (4×, 5×, 6×, 7×), *R. oligandrus* (4×, 6×) and *R. pannonicus* (3×, 4×) from the “*auricomus*” group, *R. allemannii* (4x, 5x, 6x, 8x), *R. carpaticola* (2×, 4×, 6×), *R. cassubicifolius* (2×, 4×), *R. hannae* (4×, 5×), *R. hungaricus* (4×, 5×) and *R. marginicola* (3×, 4×, 6×, 8×) from the “*cassubicus*” group and *R. kitaibelii* (3×, 4×), *R. nemorosifolius* (3×, 4×) and *R. suborbicularis* (4×, 6×) from the “*fallax*” group.

**Fig. 2:**
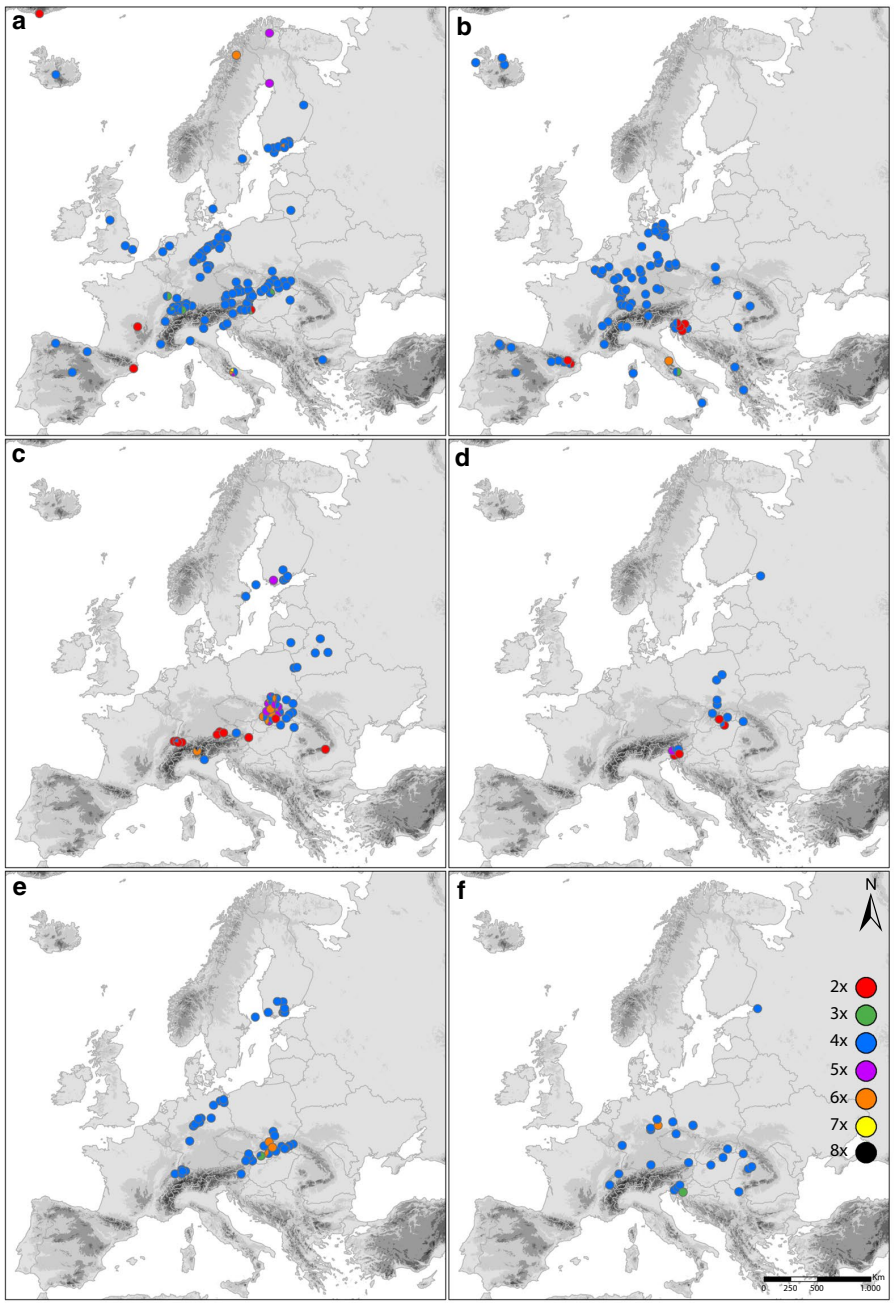
Maps showing the distribution of cytotypes of *Ranunculus auricomus* complex in Europe based on both newly acquired and previously published data. Pie charts are proportional to the frequency of a particular cytotype recorded in one population or in several closely adjacent populations (up to 20 km). **a** Previously published data for “*auricomus*” group, **b** new records for “*auricomus*” group, **c** previously published data for “*cassubicus*” group, **d** new records for “*cassubicus*” group, **e** previously published data for “fallax” group, **f** new records for “*fallax*” group

The geographic distribution of cytotypes identified in this study, which complemented previously published records, is shown in Fig. 2. Minority cytotypes (tri-, penta-, hepta- and octoploids) were found in localities where diploids and tetraploids co-occur or in close vicinity. Additionally, several cytotypes were observed in particular localities for the first time. Within the “*auricomus*” group diploid lineages were for the first time recorded in Slovenia similarly as triploids in central Italy (Fig. 2b, Additional file 1). In the “*cassubicus*” group diploids, but also a population of tetra- and pentaploids morphologically resembling the hexaploid *R. allemannii* were discovered in Slovenia (Fig. 2d, Additional file 1). Within the “*fallax*” group new records of triploids and tetraploids in Slovenia are accounted for as well as one hexaploid record in Germany (Fig. 2f, Additional file 1).

### Climatic differentiation

**Fig. 3:**
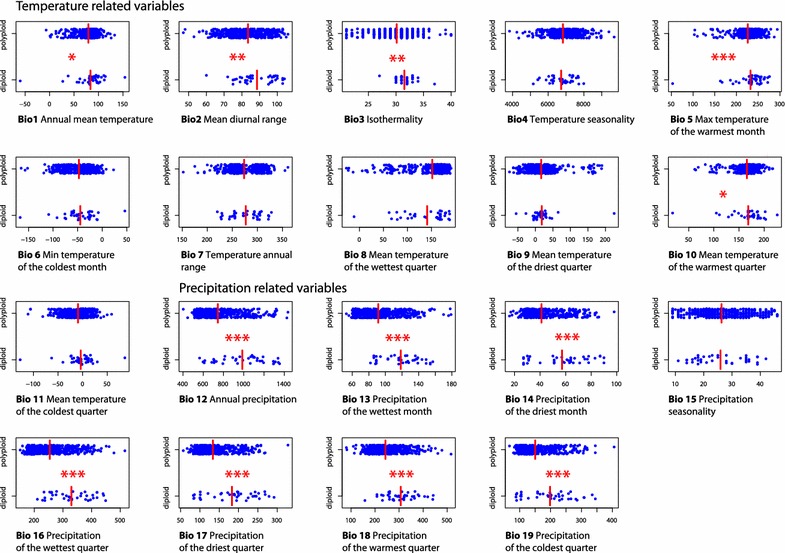
Stripchart comparisons of the climatic variables of diploid and polyploid cytotypes of *Ranunculus auricomus* complex. Asterisks indicate statistical significance based on the non-parametric Wilcoxon rank-sum test (* p ≤ 0.05, ** p ≤ 0.01, *** p ≤ 0.001), red bars indicate mean values of a particular variable and ploidy

The results of the non-parametric Wilcoxon rank-sum test of each climatic variable between diploids and polyploids are shown in Fig. 3. Highly significant differences (indicated by asterisks) were revealed for several precipitation and one temperature related variable, pointing to a certain ecological differentiation between both ploidy groups. Accordingly, polyploids tend to occupy slightly colder habitats (Bio5, Bio1) with lower precipitation (Bio12–Bio14, Bio16–Bio18).

**Fig. 4:**
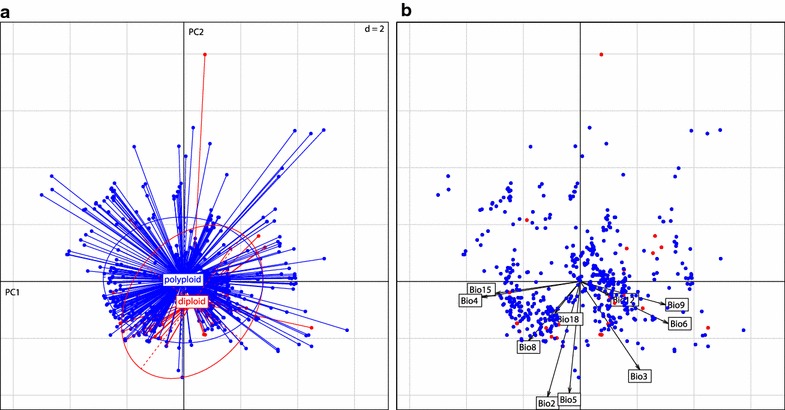
Scatterplot showing results from individual-based principal component analysis (PCA). The first two axes explain 34.64 and 22.84% of the variance among the 10 climatic variables. **a** Diploid (red) and polyploid (blue) cytotypes labelled inside the 95% inertia ellipses. **b** Biplot of objects and variables, in which arrows represent direction and magnitude of effects of environmental variables

Climatic niches of diploids and polyploids were also compared by PCA (Fig. 4). Together, the first two axes (PC1, PC2) explained 57.47% of the total variance (the first five components account for 94.20%). PC1 (explains 34.64% of the variance, Additional file 5) corresponds to a gradient in seasonality, with the temperature (Bio4) and precipitation seasonality (Bio15) and the minimal temperature of the coldest month (Bio6) and driest quarter (Bio9) showing the strongest correlations (Fig. 4b, Additional file 5). Loadings of variables for PC2 (explains 22.84% of the variance, Additional file 5) correspond to variation in temperature, being strongly correlated with mean diurnal range (Bio2) and maximal temperature of the warmest month (Bio5), (Fig. 4b, Additional file 5). A shift of 95% inertia ellipses of diploids and polyploids recovered by the PCA suggested some climatic differentiation (Fig. 4a). Significant differences in climatic variation were recovered for PC2 (W = 6019.5, p < 0.001) and PC3 (W = 15604, p < 0.001) revealing that diploids tend to occupy warmer and wetter habitats with higher mean diurnal temperature range than polyploids. However, all but one diploid were found within the climatic niche of the polyploids (Fig. 4b).

### Distribution modelling

Species distribution models are not only a good way to characterize areas suitable for the species, by using presence-background data from the study area, they are explicitly taking into account the geographically available niche space. The average Test AUCs for the four modelled groups (all diploids, all polyploids, diploid “*auricomus*” group, diploid “*cassubicus*” group) were all above 0.9, with highest values for the diploid “*cassubicus*” group (0.98). The climatic variable with the highest contribution to the models of the diploids was precipitation of warmest quarter (Bio18), the variable with the highest contribution to the polyploid models was temperature seasonality (Bio4).

**Fig. 5:**
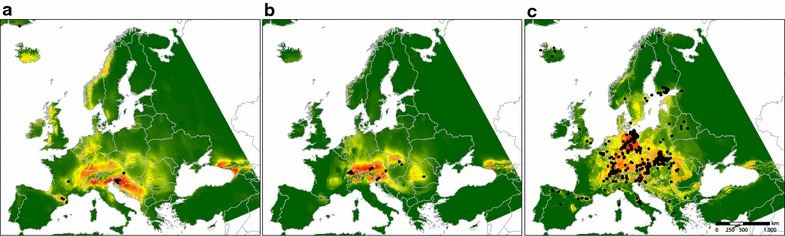
Species distribution models for diploids and polyploids of the *Ranunculus auricomus* complex based on current climate observations. Probability of occurrence is represented by different colours from low (green) to high (red). Black dots indicate current occurrence points. **a** Diploids “*auricomus*” group, current, **b** diploids “*cassubicus*” group, **c** polyploids

Present distribution patterns differ considerably between polyploids and diploids (Fig. 5). The polyploids occupy a wide range with high probabilities in Central European lowlands, while diploids of both the “*auricomus*” and the “*cassubicus*” group are more confined to the foothills of mountain ranges including the northern and southern fringe of the Alps, the Pyrenees and the Carpathians. For diploids of the “*auricomus*” group, high probabilities of occurrence were also predicted for the Balkans, and the western Caucasus including the foothills of the Caucasus on the Black Sea Coast (Colchis region) (Fig. 5a).

**Fig. 6:**
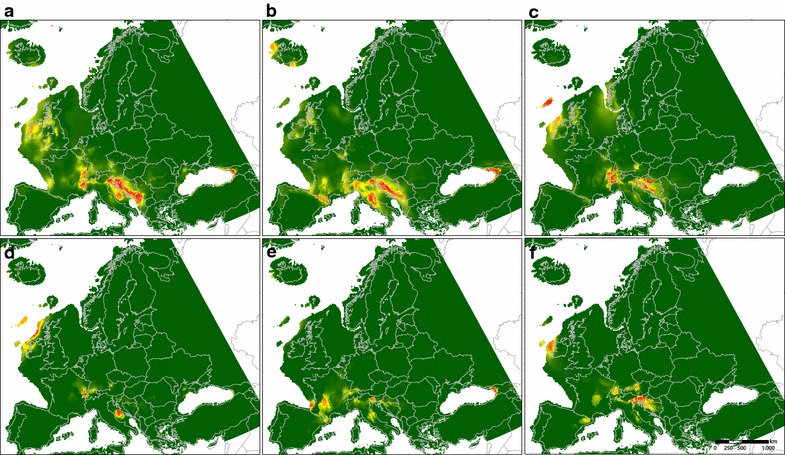
Species distribution models for diploids of the *Ranunculus auricomus* complex. Probability of occurrence is represented by different colours from low (green) to high (red). Black dots indicate current occurrence points. Results are based on the data from three different paleoclimate models representing the Last Glacial Maximum (LGM, ca. 22 kyr BP): CCSM4, MIROC-ESM and MPI ESM-P. **a** Diploids “*auricomus*” group, CCSM4; **b** diploids “*auricomus*” group, MPI ESM-P; **c** diploids “*auricomus*” group, MIROC-ESM; **d** diploids “*cassubicus*” group, CCSM4; **e** diploids “*cassubicus*” group, MPI ESM-P; **f** diploids “*cassubicus*” group, MIROC-ESM

Modelled past distributions of the “*auricomus*” group show highest probabilities of occurrence in the western foothills of the Alps, in southern foothills of the Alps stretching into the Balkans and the Colchis region (Fig. 6a–c). For the “*cassubicus*” group, there are only small areas with high probabilities of occurrence, mainly in the West and South of the Alps (Fig. 6d–f).

## Discussion

Chromosome number and ploidy level are among the key genomic variables of plant species. These data accumulated over the last 80 years and represent a valuable resource to study the link of polyploidy and geographic distribution. In this study we combine new and previously published ploidy data from a continent wide distribution range as well as main evolutionary lineages of the apomictic *R. auricomus* complex in order to disentangle the cytogeographic patterns in this taxonomic group using descriptive statistical and modelling approaches.

### Relative genome size of diploid lineages

For the first time genome sizes among diploid lineages of the *R. auricomus* complex were compared. We assume that the relative genome size measured using AT-specific DAPI truly represents the absolute genome size because the GC-content in the genus *Ranunculus* is well conserved [47]. Significant differences in relative genome size on the diploid level were observed between the “*auricomus*” and “*cassubicus*” groups. The most likely cause of genome size divergence between closely related species, independent of change in chromosome number, is the transposable elements (TEs) dynamics [48, 49]. It has been hypothesized that the differences in TE abundance originate from differential rates of DNA elimination/proliferation through recombination [50, 51] or due to highly efficient epigenetic silencing pathways [52]. For instance, it has been shown that the Tekay chromoviral elements are relatively abundant in the genomes of *Anemone* s.l. (Ranunculaceae) and are probably contributing to genome evolution and speciation in the whole family [53]. Divergent relative genome sizes are in line with the dated split of the diploid *R. carpaticola* from the “*cassubicus*” group and diploid *R. notabilis* from the “*auricomus*” group, which has been estimated for ca. 900 kyr BP. In contrast the split between closely related diploid sister taxa *R. carpaticola* and *R. cassubicifolius* (both “*cassubicus*” group) was estimated for ca. 315 kyr BP [30]. Hence, lineages belonging to different, more distantly related groups had also more time to accumulate differential TE bulks than more closely related taxa similarly as reported for genus *Oryza* [54]. Insignificant differences in genome sizes within the “*cassubicus*” group were also already demonstrated for *R. carpaticola* and *R. cassubicifolius* (1C values of 2.93–3.05 pg and 3.11 pg, respectively; [22]).

### Geographic distribution of diploid lineages

Records on diploids from the *R. auricomus* complex are rare and restricted to the “*auricomus*” and “*cassubicus*” groups and to particular geographical areas. In the “*auricomus*” group diploids were previously found in the Massif Central, Pyrenees and Austria [55–57]. Diploidy was also reported from Greenland [58], which in our view needs confirmation. In the “*cassubicus*” group diploids were recorded in Alpine Foreland, the Carpathians and Austria [33, 57, 59–61]. In scope of this study ten diploid populations belonging to about six taxa (*R. cassubicifolius* and five yet undescribed taxa from the “*auricomus*” group) were discovered in Slovenia and Hungary. The Hungarian diploid populations of the “*cassubicus*” group and one Slovenian taxon were found in similar habitats (i.e. beech, hornbeam and oak forests) as previously reported for the Carpathian taxon [32]. The remainder of the Slovenian diploid populations were found in meadows and willow shrub edges.

Climatic variables suggest that the diploids are distributed in climatically diverse habitats. However, all of these localities have been suggested as extra-Mediterranean glacial refugia for temperate species [62]. It is widely accepted that the distribution of diploids in the polyploid species complexes corresponds with putative Pleistocene refugia [7, 63]. Hence, the southeastern parts of Alps and northwestern Dinaric Alps (i.e. Slovenia) were proposed as refugia for beech (*Fagus sylvatica* L.) [64, 65]. Refugia in Western and Eastern Carpathians are suggested for several other temperate tree species [66–68], including hornbeam (*Carpinus betulus* L.), with which diploids from the *R. auricomus* complex are closely associated [32]. Similarly, refugia for species associated with temperate forests were also identified in the Eastern Pyrenees [69, 70] and Alpine Foreland [71].

This is substantiated by distribution modelling using the paleoclimate data from the LGM (ca. 22 kyr BP). Predicted diploid distribution areas, where diploids are also currently present, were given the highest probabilities in most of the three models under paleoclimatic scenarios. This implies that most of the diploids remained restricted to their glacial refugia and did not migrate or significantly expanded their distribution range. This phenomenon was observed in several other polyploid complexes and is explained by a certain environmental stability due to a climatic and topographic heterogeneity in the refugia [72–74]. It is hypothesized that in particular refugia, which are close to high mountain ranges, a high diversity of ecological niches is present, which offers species the opportunity to migrate along the altitudinal gradient, rather than the latitudinal one [75].

Interestingly, paleoclimatic models did not recover Austria and the Carpathians as areas with high probability of occurence during LGM. On one hand, in relatively steep elevation and climatic gradient represented by mountain areas, the species may have survived in microclimatic refugia not well represented in the rather coarse macroclimatic data that were employed for distribution modelling. Alternatively, Austria and the Carpathians may represent areas which were colonized later from the Balkans. This scenario has been recently reconsidered for *Alnus glutinosa*, suggesting that the Carpathians were colonized from the Balkan Peninsula 7.9–25.5 kyr BP in the pre-Late Pleniglacial period [76]. *Alnus glutinosa* represent a major element of temperate riverine forests, in which members of the *R. auricomus* complex are often found. The time period of our LGM model (ca. 22 kyr BP) fits the lower time bound of the colonization estimation, which in our view supports the secondary colonization of Austria and the Carpathians.

Distribution modelling approaches with both current and paleoclimate data identified the foothills of the Caucasus on the Black Sea Coast (Colchis region) as a potential distribution area of the diploids. In fact, this area is also recognized as a Pleistocene refugium due to the presence of several Tertiary relict species and relict forest communities [77, 78] and there are records of the *R. auricomus* complex from humid temperate forests of the Caucasus [17].

Diploids from the “*auricomus*” and “*cassubicus*” groups were for the first time found in sympatry. Nevertheless it seems that gene flow between these lineages is restricted, as suggested by discrete intervals of relative genome sizes. Diploids from the *R. auricomus* complex were reported to be self-incompatible [79] and mentor effects [i.e. breakdown of the self-incompatibility (SI) system] induced by pollen from polyploid apomicts are limiting the introgression of apomixis into sexual species [22]. Hence, self-incompatibility may also apply for diploid lineages separated for 900 kyr, in which certain allelic composition or the genetic distance causes the breakdown of SI systems and limits ongoing hybridization. However, to be fully conclusive, experimental crosses with detailed progeny evaluation need to be carried out.

### Relative genome size of polyploids

In tetraploids the interval of relative genome size is broader than that of diploids and represents a continuous range, with an exception of the sample Du-31159. This fact is in line with the presumed origin of the polyploid lineages by hybridizations of sexual ancestors from different groups with subsequent polyploidizations and genome rearrangements as well as rare introgressions [22, 28]. Non-linear increase of DNA content was previously also observed in the tetraploid *R. hungaricus*, which suggests that this taxon is not a derivative of the sexual *R. cassubicifolius* and *R. carpaticola* alliance alone [27]. On the other hand, a reduction of DNA content was observed in the autotetraploid and hexaploid hybrid derivatives of diploid *R. cassubicifolius* [24]. Moreover, the genomes of polyploid apomictic lineages seem to diversify faster in comparison with their sexual progenitors [30, 31], which may additionally add to the relative genome size variation.

### Geographic distribution of polyploids

Ecological differentiation among cytotypes is an extensively discussed topic (e.g., [8, 80]) and implies reproductive isolation and neutral or adaptive processes. Polyploids in the *R. auricomus* complex are reproducing mostly via apomixis [18, 20, 22, 81, 82], whereas diploids are reproducing sexually [23, 30, 82, 83]. Hence, the larger distribution of polyploids can be to great extent explained by superior colonizing abilities due to uniparental reproduction also referred to as Baker’s law [12]. However, the phenomenon of “geographical parthenogenesis” is certainly more complex [14, 84], which is also indicated in our data. Polyploidization, and in particular allopolyploidization, is often considered advantageous when colonizing formerly glaciated areas due to possibly favourable genetic rearrangements which accompany the genome doubling [3, 85]. Based on comparisons of the climatic preferences *R. auricomus*-polyploids occupy slightly drier and colder habitats than the diploids. Likewise, the species distribution models show broader distributions/niches for the polyploids, but with considerable overlap with diploids. Hence, similarly as recently observed in *R. kuepferi* [86], the diploid vs polyploid distribution pattern in *R. auricomus* complex might result from a combination of different factors. In a sympatric range (recently or during last glaciation in refugia), polyploids might have been restricted to marginal parts of the diploid niche, which could have directed the further evolution of the polyploid niche towards slightly cooler and drier conditions in the allopatric range. Pre-adapted polyploid genotypes might have consequently spread through apomictic mode of reproduction, which resulted in a broader, but climatically not much diverged, distribution range. Interestingly, a snapshot of these processes was also observed recently. In *R. carpaticola* sexual diploids were recorded almost exclusively in forest habitats and polyploid apomicts showed a tendency to inhabit artificial meadows, representing possibly cooler (in winter) and drier conditions [32].

## Conclusions

Comparison of current and three paleodistribution models suggests that diploid sexuals from *R. auricomus* complex remained restricted to their glacial refugia and did not migrate or significantly expanded their distribution range. In contrast, mostly apomictic polyploids have larger distribution and occupy slightly drier and colder habitats than the sexual diploid ancestors. The change of reproductive mode and selection due to competition with the diploids may have facilitated the shift of climatic preferences in polyploids. Much broader distribution of polyploid apomicts may have been consequently achieved due to faster colonization mediated by uniparental reproductive system.

## Additional files

**Additional file 1.** List of studied accessions. “Sample/standard ratio” refers to relative genome size as described in “Methods”. Group: aur—“*auricomus*” group, cass—“*cassubicus*” group, fal—“*fallax*” group. Sample number is identical with the collection number of the herbarium specimen. Herbarium codes follow Index Herbariorum (http://sweetgum.nybg.org/science/ih/).

**Additional file 2.** Relative genome size and derived DNA-ploidy in studied accessions of the *Ranunculus auricomus* complex with microphotograph of the somatic chromosomes (2n = 2× = 16) of *Ranunculus austroslovenicus* ined., Du-30442. Relative genome size is expressed as a ratio of the sample and the internal reference standard (*P. sativum*). Each circle represents an analysed sample; full circles represent accessions for which chromosome numbers were determined.

**Additional file 3.** List of previously published chromosome counts from the *Ranunculus auricomus* complex in Europe. Spreadsheet “Data” lists extracted taxa, localities and chromosome numbers. Group: aur—“*auricomus*” group, cass—“*cassubicus*” group, fal—“*fallax*” group, ?—could not have been determined. Remaining captions are self-explanatory. Spreadsheet “References” lists reviewed literature.

**Additional file 4.** Correlation coefficients between 19 climatic variables extracted for the *Ranunculus auricomus* complex. Bold font highlights the absolute values greater than 0.8. Variables in bold were removed in the PCA analysis and for the distribution modelling.

**Additional file 5.** Loadings of variables, proportion and cumulative proportion of the variance of the first 5 PCA axes on a set of 10 climatic variables extracted for *Ranunculus auricomus* complex. Bold font highlights three most extreme values of loadings for particular axes.
