# Supplementary figures and images for: Climatic differentiation in polyploid apomictic Ranunculus auricomus complex in Europe
Source: BMC Ecol. 2018 May 21;18:16. doi: 10.1186/s12898-018-0172-1 (PMC5963127; doi:10.1186/s12898-018-0172-1)

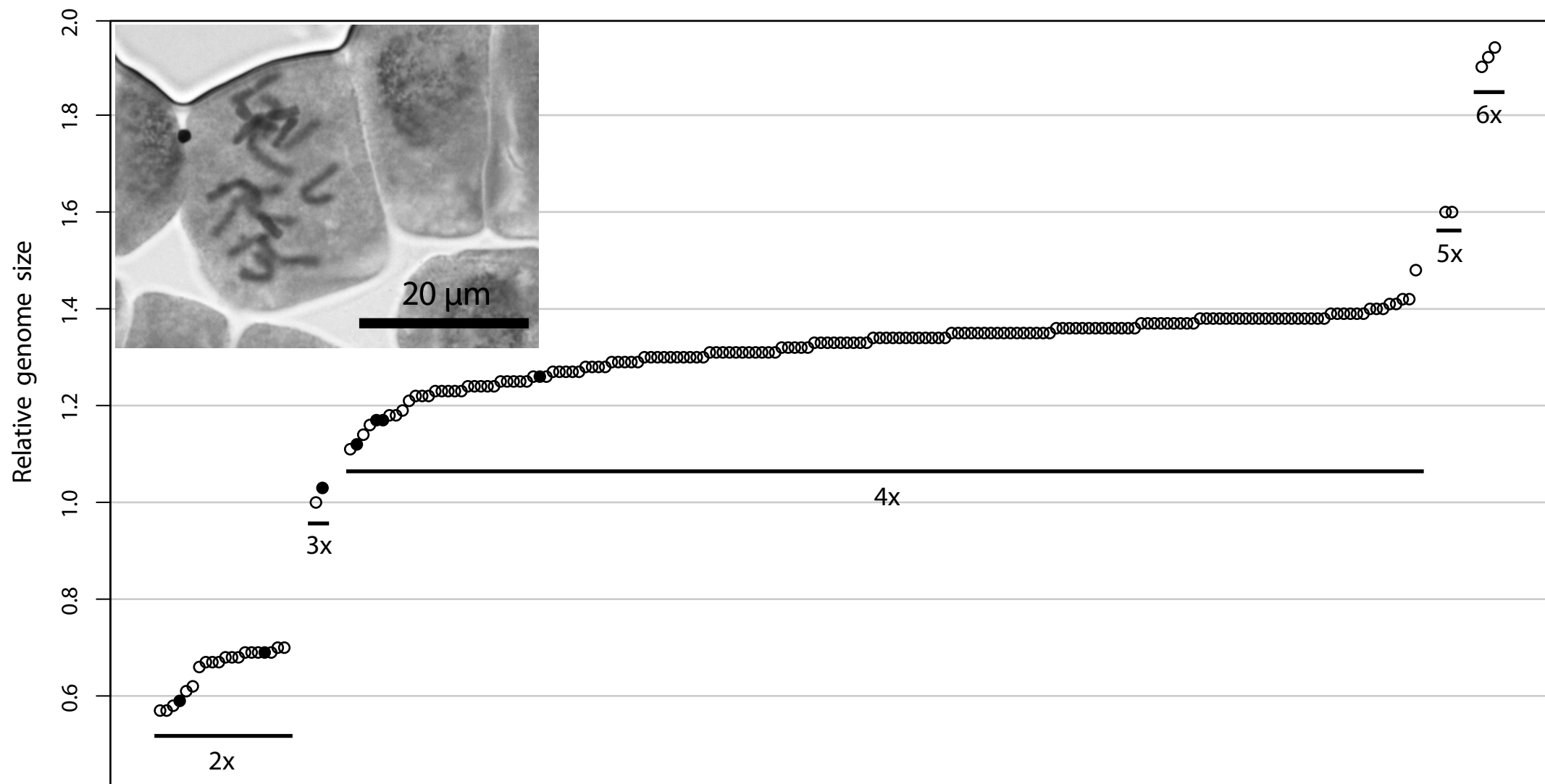

Supplement: Supplementary file 2 — Additional file 2. Relative genome size and derived DNA-ploidy in studied accessions of the Ranunculus auricomus complex with microphotograph of the somatic chromosomes (2n = 2× = 16) of Ranunculus austroslovenicus ined., Du-30442. Relative genome size is expressed as a ratio of the sample and the internal reference standard (P. sativum). Each circle represents an analysed sample; full circles represent accessions for which chromosome numbers were determined. [file 12898_2018_172_MOESM2_ESM.pdf]
