# Supplementary material for: Climatic differentiation in polyploid apomictic Ranunculus auricomus complex in Europe
Source: BMC Ecol. 2018 May 21;18:16. doi: 10.1186/s12898-018-0172-1 (PMC5963127; doi:10.1186/s12898-018-0172-1)
Supplement: Supplementary file 4 — Additional file 4. Correlation coefficients between 19 climatic variables extracted for the Ranunculus auricomus complex. Bold font highlights the absolute values greater than 0.8. Variables in bold were removed in the PCA analysis and for the distribution modelling. [file 12898_2018_172_MOESM4_ESM.docx]

**Additional file 4** Correlation coefficients between 19 climatic variables extracted for the *Ranunculus auricomus* complex. Bold font highlights the absolute values greater than 0.8. Variables in bold were removed in the PCA analysis and for the distribution modelling.

|  | **Bio1** | Bio2 | Bio3 | Bio4 | Bio5 | Bio6 | **Bio7** | Bio8 | Bio9 | **Bio10** | **Bio11** | Bio12 | **Bio13** | **Bio14** | Bio15 | **Bio16** | **Bio17** | Bio18 | **Bio19** |
| --- | --- | --- | --- | --- | --- | --- | --- | --- | --- | --- | --- | --- | --- | --- | --- | --- | --- | --- | --- |
| Bio1 | X |  |  |  |  |  |  |  |  |  |  |  |  |  |  |  |  |  |  |
| Bio2 | 0.358 | X |  |  |  |  |  |  |  |  |  |  |  |  |  |  |  |  |  |
| Bio3 | 0.468 | 0.567 | X |  |  |  |  |  |  |  |  |  |  |  |  |  |  |  |  |
| Bio4 | -0.168 | 0.308 | -0.587 | X |  |  |  |  |  |  |  |  |  |  |  |  |  |  |  |
| Bio5 | **0.844** | 0.628 | 0.258 | 0.334 | X |  |  |  |  |  |  |  |  |  |  |  |  |  |  |
| Bio6 | **0.776** | -0.127 | 0.514 | -0.695 | 0.354 | X |  |  |  |  |  |  |  |  |  |  |  |  |  |
| Bio7 | -0.043 | 0.629 | -0.273 | **0.923** | 0.479 | -0.651 | X |  |  |  |  |  |  |  |  |  |  |  |  |
| Bio8 | 0.369 | 0.285 | -0.061 | 0.415 | 0.492 | 0.028 | 0.373 | X |  |  |  |  |  |  |  |  |  |  |  |
| Bio9 | 0.472 | -0.049 | 0.427 | -0.568 | 0.205 | 0.672 | -0.464 | -0.377 | X |  |  |  |  |  |  |  |  |  |  |
| Bio10 | **0.887** | 0.441 | 0.149 | 0.293 | **0.968** | 0.450 | 0.364 | 0.529 | 0.223 | X |  |  |  |  |  |  |  |  |  |
| Bio11 | **0.864** | 0.077 | 0.618 | -0.631 | 0.493 | **0.971** | -0.512 | 0.059 | 0.680 | 0.556 | X |  |  |  |  |  |  |  |  |
| Bio12 | -0.115 | 0.099 | 0.246 | -0.265 | -0.158 | -0.001 | -0.128 | -0.442 | 0.080 | -0.253 | 0.021 | X |  |  |  |  |  |  |  |
| Bio13 | -0.216 | 0.266 | 0.207 | -0.071 | -0.146 | -0.233 | 0.100 | -0.322 | -0.091 | -0.280 | -0.171 | **0.903** | X |  |  |  |  |  |  |
| Bio14 | 0.007 | 0.006 | 0.298 | -0.376 | -0.119 | 0.194 | -0.279 | -0.383 | 0.154 | -0.177 | 0.179 | **0.920** | 0.716 | X |  |  |  |  |  |
| Bio15 | -0.171 | 0.410 | -0.080 | 0.460 | 0.096 | -0.517 | 0.564 | 0.231 | -0.361 | 0.015 | -0.390 | -0.235 | 0.151 | -0.509 | X |  |  |  |  |
| Bio16 | -0.203 | 0.247 | 0.181 | -0.058 | -0.134 | -0.222 | 0.100 | -0.311 | -0.104 | -0.259 | -0.166 | **0.914** | **0.989** | 0.721 | 0.130 | X |  |  |  |
| Bio17 | 0.010 | -0.018 | 0.311 | -0.418 | -0.135 | 0.223 | -0.320 | -0.420 | 0.215 | -0.190 | 0.205 | **0.918** | 0.703 | **0.992** | -0.536 | 0.70**8** | X |  |  |
| Bio18 | -0.201 | 0.310 | 0.144 | 0.084 | -0.095 | -0.304 | 0.208 | 0.034 | -0.389 | -0.207 | -0.252 | 0.774 | **0.876** | 0.632 | 0.165 | **0.890** | 0.597 | X |  |
| Bio19 | -0.037 | -0.089 | 0.285 | -0.467 | -0.196 | 0.227 | -0.372 | -0.606 | 0.410 | -0.247 | 0.205 | **0.866** | 0.645 | **0.895** | -0.509 | 0.650 | **0.917** | 0.403 | X |

Bio1 Annual mean temperature Bio15 Precipitation seasonality

Bio2 Mean diurnal range Bio16 Precipitation of the wettest quarter

Bio3 Isothermality Bio17 Precipitation of the driest quarter

Bio4 Temperature seasonality Bio18 Precipitation of the warmest quarter

Bio5 Max temperature of the warmest month Bio19 Precipitation of the coldest quarter

Bio6 Min temperature of the coldest month

Bio7 Temperature annual range

Bio8 Mean temperature of the wettest quarter

Bio9 Mean temperature of the driest quarter

Bio10 Mean temperature of the warmest quarter

Bio11 Mean temperature of the coldest quarter

Bio12 Annual precipitation

Bio13 Precipitation of the wettest month

Bio14 Precipitation of the driest month
