# Supplementary material for: Climatic differentiation in polyploid apomictic Ranunculus auricomus complex in Europe
Source: BMC Ecol. 2018 May 21;18:16. doi: 10.1186/s12898-018-0172-1 (PMC5963127; doi:10.1186/s12898-018-0172-1)
Supplement: Supplementary file 5 — Additional file 5. Loadings of variables, proportion and cumulative proportion of the variance of the first 5 PCA axes on a set of 10 climatic variables extracted for Ranunculus auricomus complex. Bold font highlights three most extreme values of loadings for particular axes. [file 12898_2018_172_MOESM5_ESM.docx]

**Additional file 5** Loadings of variables, proportion and cumulative proportion of the variance of the first 5 PCA axes on a set of 10 climatic variables extracted for *Ranunculus auricomus* complex. Bold font highlights three most extreme values of loadings for particular axes.

| **Variable** | **Description** | **PC1** | **PC2** | **PC3** | **PC4** | **PC5** |
| --- | --- | --- | --- | --- | --- | --- |
| Bio2 | Mean diurnal range | -0.161 | **-0.570** | 0.139 | 0.299 | 0.129 |
| Bio3 | Isothermality | 0.295 | **-0.437** | 0.176 | 0.103 | **0.513** |
| Bio4 | Temperature seasonality | **-0.487** | -0.078 | -0.108 | 0.115 | **-0.404** |
| Bio5 | Max temperature of the warmest month | -0.054 | **-0.552** | -0.255 | 0.005 | **-0.453** |
| Bio6 | Min temperature of the coldest month | **0.436** | -0.208 | -0.224 | -0.283 | -0.057 |
| Bio8 | Mean temperature of the wettest quarter | -0.256 | -0.292 | **-0.282** | **-0.618** | 0.178 |
| Bio9 | Mean temperature of the driest quarter | **0.422** | -0.115 | -0.145 | **0.409** | -0.275 |
| Bio12 | Annual precipitation | 0.151 | -0.071 | **0.603** | -0.093 | **-0.404** |
| Bio15 | Precipitation seasonality | -0.420 | -0.059 | 0.063 | **0.379** | 0.270 |
| Bio18 | Precipitation of the warmest quarter | -0.124 | -0.154 | **0.595** | -0.324 | -0.073 |
|  |  |  |  |  |  |  |
|  | Proportion of variance [%] | 34.64 | 22.84 | 21.51 | 8.71 | 6.51 |
|  | Cumulative proportion [%] | 34.64 | 57.47 | 78.98 | 87.70 | 94.21 |
